# Suboptimal peak inspiratory flow, COPD exacerbations, hospitalisations and disease burden: a longitudinal follow-up in a multicentre real-world study

**DOI:** 10.1183/23120541.00343-2026

**Published:** 2026-11-09

**Authors:** Valeria Perugini, Chin Kook Rhee, Ji-Yong Moon, Pei Yee Tiew, Seung Won Ra, Pietro Pirina, Kwang Ha Yoo, Bernardino Alcázar Navarrete, Caroline Gouder, Virginia Almadana Pacheco, Annie Navarro-Rolon, Matevž Harlander, Thérèse S. Lapperre, Chee Hong Loh, Elsa Naval, Marc Miravitlles, Omar Usmani

**Affiliations:** 1 Respiratory Effectiveness Group, Ely, UK; 2 Division of Pulmonary, Allergy, and Critical Care Medicine, Department of Internal Medicine, Konkuk University Medical Center, Konkuk University School of Medicine, Seoul, Korea; 3 Department of Respiratory and Critical Care Medicine, Singapore General Hospital, Singapore, Singapore; 4 Department of Internal Medicine, Ulsan University Hospital, University of Ulsan College of Medicine, Ulsan, Republic of Korea; 5 Department of Medicine, Surgery and Pharmacy, University of Sassari, Sassari, Italy; 6 Respiratory Department, Hospital Universitario Virgen de las Nieves, Granada, Spain; 7 Universidad de Granada, Granada, Spain; 8 CIBER de Enfermedades Respiratorias (CIBERES), Granada, Spain; 9 Department of Respiratory Medicine, Mater Dei Hospital, Msida, Malta; 10 Respiratory Department, Hospital Universitario Virgen Macarena, Seville, Spain; 11 Department of Pneumology, Fundació Hospital Sant Joan de Déu de Martorell, Martorell, Spain; 12 Institute for Research and Innovation in Life and Health Sciences in Central Catalonia (IRIS-CC), Central Catalonia, Spain; 13 Department of Pulmonary Diseases, University Medical Centre, Ljubljana, Slovenia; 14 Faculty of Medicine, University of Ljubljana, Ljubljana, Slovenia; 15 Department of Pulmonary Medicine, University Hospital Antwerp, Antwerp, Belgium; 16 Laboratory of Experimental Medicine and Pediatrics, University of Antwerp, Antwerp, Belgium; 17 Respiratory and Critical Care Medicine, Changi General Hospital, Singapore; 18 Pneumology Service, Hospital Universitario de La Ribera, Alzira, Spain; 19 Pneumology Department, Hospital Universitari Vall d'Hebron, Vall d'Hebron Institut de Recerca, Vall d'Hebron Barcelona Hospital Campus, Barcelona, Spain; 20 CIBERES, Barcelona, Spain; 21 National Heart and Lung Institute, Imperial College London, London, UK; 22 Royal Brompton Hospital and St Mary's Hospital London, London, UK; 23 These authors contributed equally

## Abstract

**Background:**

Peak inspiratory flow (PIF) is a critical determinant of dry powder inhaler (DPI) effectiveness in managing patients with COPD. Suboptimal PIF may compromise drug delivery and clinical outcomes. The objective of the present study was to evaluate the association between repeated PIF measures and disease exacerbations, hospitalisations, and symptom burden in patients with stable COPD.

**Methods:**

In this multi-country, observational cohort, 415 participants aged ≥40 years with confirmed COPD and a smoking history underwent PIF measurements at their prescribed inhaler's resistance, and low- and high-resistance levels using the In-Check DIAL G16. Clinical assessments occurred at 3 months (follow-up 1 (FU1)) and 6 months (follow-up 2 (FU2)) including spirometry, COPD Assessment Test scores, medication use and exacerbation/hospitalisation rates.

**Results:**

At FU1, 29.4% (n=113/384) had typical suboptimal PIF and 28.6% (n=108/377) remained suboptimal at FU2. Participants with suboptimal PIF were more often female and current smokers, with consistently poorer lung function across both visits. Lower forced expiratory volume in 1 s (33.7±9.1% pred *versus* 45.7±9.2% pred, p=0.01), actual forced vital capacity and inspiratory capacity were observed. Suboptimal PIF significantly predicted moderate (p=0.03) and severe (p=0.01) exacerbations, was associated with higher hospitalisation risks (p=0.05) and with greater use of short-acting beta agonist, oral corticosteroid and triple therapy.

**Conclusions:**

Suboptimal PIF is a stable, clinically meaningful marker of worse COPD outcomes and may guide more personalised inhaler selection.

## Shareable abstract

**Suboptimal peak inspiratory flow (PIF) remains common in COPD over time and is associated with suboptimal inhaler use, highlighting the need for routine PIF assessment to support personalised inhaler selection** https://bit.ly/3SjsTLg

## Introduction

Chronic obstructive pulmonary disease (COPD) is a progressive respiratory disorder characterised by persistent respiratory symptoms and airflow limitation stemming from airway and/or alveolar abnormalities [1]. A hallmark of COPD is the occurrence of exacerbations, which significantly impair quality of life and overall health [2]. Effective management is thus essential to alleviate daily symptoms, slow disease progression and reduce exacerbation frequency and severity [3].

Recent developments in personalised medicine aim to improve COPD management by tailoring therapeutic interventions to individual patient characteristics, including peak inspiratory flow (PIF) and inhaler choice [4]. These personalised approaches aim to optimise medication delivery and effectiveness based on each patient's specific needs and capabilities [5].

PIF, the maximum speed of inhalation through a device, is critical for effective drug delivery [6] and particularly relevant for dry powder inhalers (DPIs), which require sufficient inspiratory flow matched to device resistance to aerosolise medication [7]. When PIF falls below this threshold, drug delivery is compromised, leading to poor symptom control and increased exacerbation risk [8]. Assessing PIF is therefore essential to ensuring patients derive maximum benefit from their inhaled therapies [9].

Inhaler choice is equally important, as devices differ in design, mechanics and required inspiratory effort [10] and may influence both drug delivery and treatment adherence [11]. Selecting an inhaler that matches a patient's physical capabilities and preferences is vital for effective COPD management [12, 13]. Understanding the interaction between PIF and inhaler choice is thus essential for tailoring COPD treatment to individual needs and optimising clinical outcomes, including reducing exacerbations [14].

This study investigates optimal and suboptimal PIF across inhaler device type and investigates the association of suboptimal PIF with lung function, exacerbations, hospitalisations and symptom burden in COPD.

## Methods

### Study design and study population

This was an observational, multi-centre, prospective, real-life cohort study designed to assess the relationship between PIF and inhaler device type in predicting exacerbations and managing symptom burden in patients with COPD. The study was conducted across 17 international centres located in five European countries (Spain, Malta, Italy, Belgium and Slovenia), as well as South Korea and Singapore.

The study protocol was registered on public databases before initiation (clinicalTrials.gov NCT04360226 Encepp EUPAS34689) and approved by local ethics committees in each participating country. All participants provided written informed consent.

This manuscript reports data from two follow-up visits conducted at 3 months (follow-up 1 (FU1)) and 6 months (follow-up 2 (FU2)) post-baseline, using the same methodology as the baseline assessment, with findings reported separately [15].

Eligible participants were aged ≥40 years with a confirmed COPD diagnosis according to the 2024 Global Initiative for Chronic Obstructive Lung Disease directive [16]. Additional inclusion criteria required a smoking history of at least 10 pack-years (current or former smokers), stable disease with no exacerbations in the preceding 4 weeks, the ability to perform serial lung function tests and at least 6 months of prescribed inhaled medication.

Exclusion criteria included the inability to provide informed consent, concurrent participation in other clinical trials involving COPD medications, a recent exacerbation within the past 4 weeks or the presence of any life-threatening disease with a life expectancy of less than 6 months.

Based on these criteria, 415 participants were enrolled. Of these, 384 completed the FU1 visit and 377 remained in the study through the FU2. The distribution of inhaler devices used by participants at each follow-up is summarised in table 1.

**TABLE 1:** Distribution of inhaler devices at 3-month follow-up 1 (FU1) and 6-month follow-up 2 (FU2) visits, reported as n (%)

Inhaler device	FU1 N=384	FU2 N=377
**pMDI, n (%)**	68 (17.7)	65 (17.2)
**SMI, n (%)**	71 (18.5)	69 (18.3)
**DPI, n (%)**	245 (63.8)	243 (64.4)

DPI: dry powder inhaler; pMDI: pressurised metered-dose inhaler; SMI: soft mist inhaler.

#### Definition of exacerbations

Exacerbations were classified as follows:

- Moderate exacerbations: defined as requiring an acute course of antibiotics or oral steroids for COPD.
- Severe exacerbations: defined as requiring emergency room visits or hospitalisation for more than 24 h due to COPD.

The frequency and severity of exacerbations during the 6 months preceding each visit were documented based on both medical records and patient-reported data. At FU1, this 6-month recall period partially overlapped with the baseline assessment window, whereas at FU2 it primarily reflected the period following FU1. As such, exacerbation data represent repeated assessments of recent exacerbation history rather than strictly nonoverlapping prospective intervals.

### PIF measurements

PIF was measured using the In-Check DIAL G16 device (Clement Clarke International Ltd, Harlow, UK). This specialised PIF meter, fitted with disposable mouthpieces and an adjustable dial, simulates the resistance (R) characteristics of various inhaler types. Resistance levels ranged from R0 (no resistance, simulating pressurised metered-dose inhalers (pMDIs) and soft mist inhalers (SMIs)), to R5 (high resistance for DPIs), each corresponding to the minimum PIF needed for specific inhaler types. Device resistance settings were matched to each participant's prescribed inhaler. The distribution of participants across the different resistance levels (R0–R5), along with their PIF status (optimal or suboptimal), is summarised in table 2.

**TABLE 2:** Participant distribution across inhaler device resistance levels and corresponding peak inspiratory flow (PIF) status at follow-up 1 (FU1) and follow-up 2 (FU2)

	FU1			FU2		
In-check device resistance (R)	Total participants N=384 (100%)	Optimal PIF n=271 (70.6%)	Suboptimal PIF n=113 (29.4%)	Total participants N=377 (100%)	Optimal PIF n=269 (71.4%)	Suboptimal PIF n=108 (28.6%)
**pMDIs/SMI (R0) (n, %)**	139 (36.2)	88 (32.5)	51 (45.1)	134 (35.5)	87 (32.3)	47 (43.5)
**Low (R1) (n, %)**	51 (13.3)	40 (14.8)	11 (9.7)	51 (13.5)	39 (14.5)	12 (11.1)
**Low–medium (R2) (n, %)**	142 (37)	108 (39.9)	34 (30.1)	140 (37.1)	107 (39.8)	33 (30.6)
**Medium (R3) (n, %)**	34 (8.9)	23 (8.5)	11 (9.7)	33 (8.8)	22 (8.2)	11 (10.2)
**Medium-high (R4) (n, %)**	7 (1.8)	5 (1.8)	2 (1.8)	8 (2.1)	7 (2.6)	1 (1)
**High (R5) (n, %)**	11 (2.9)	7 (2.6)	4 (3.5)	11 (2.9)	7 (2.6)	4 (3.7)

Note: Percentages for optimal and suboptimal PIF are calculated within each PIF group (column-wise), using the total number of participants in each group as the denominator. pMDI: pressurised metered-dose inhaler; SMI: soft mist inhaler.

PIF was measured in three different ways at FU1, as follows: 1) typical PIF, defined as the inspiratory flow generated when participants were asked to inhale in their usual, at-home manner, without additional coaching, measured at the resistance level specific to the participant's prescribed inhaler device; 2) PIF at low resistance, measured at the lowest (R0) resistance setting; and 3) PIF at high resistance, measured at the highest (R5) resistance setting [17]. At each resistance setting, three inhalation manoeuvres were performed and the highest value was used for analysis, in line with standard PIF assessment practice to ensure reproducibility.

For participants using more than one maintenance inhaler, DPI measurements were prioritised. In cases where participants used both a pMDI/SMI and a DPI, or two DPIs with different resistance levels, measurements were taken at the higher DPI resistance level.

Inspiratory capacity was recorded as a standalone measure during spirometry and was not calculated from forced vital capacity (FVC) or forced expiratory volume in 1 s (FEV_1_). Inspiratory capacity and FVC were recorded in litres (L) only, as % predicted values were not consistently available across participating centres due to differences in reference equations in this multi-country study and to maintain comparability across sites.

To determine if inadequate PIF was related to physiological limitation or inhalation technique was a limiting factor, at FU1, additional PIF measurements were taken as participants inhaled in their typical, at-home manner. Participants using DPIs (R1 to R5 resistance settings) were instructed to exhale to end-tidal breath and then inhale rapidly and forcefully through their device. Participants using pMDIs or SMIs (R0 resistance setting) were instructed to inhale gently and slowly.

Based on their typical PIF and their low- and high-resistance effort PIF measurements, participants were categorised as follows:

- Can do: typical PIF met or in the case of DPIs successfully exceeded the optimal PIF required for their inhaler.
- Can, but won't do: participants who were capable of achieving optimal PIF under instructed conditions, but whose typical PIF during habitual inhalation fell outside the optimal range for their device.
- Cannot do: unable to achieve the optimal PIF required for their inhalers.

Suboptimal PIF in DPIs was characterised by a typical PIF falling below the device-specific threshold and was assessed at both FU1 and FU2 [17]. Suboptimal PIF in pMDIs was characterised by a typical PIF exceeding the optimal threshold at both FU1 and FU2.

### Data collection and management

Participant information was recorded confidentially in a clinical data management system *via* an electronic case report form, completed by investigators at each study centre. All investigators received training and individualised access rights to the system.

Data entries were routinely reviewed by a data manager (Bioclever, Barcelona, Spain) to ensure accuracy and consistency. This review included both automated logical checks and manual evaluations to verify data accuracy, management and secure storage.

### Statistical analysis

The study analysis followed the Strengthening the Reporting of Observational Studies in Epidemiology (STROBE) criteria [18]. Statistical analyses were conducted using R software, an open-source program based on the S language.

Qualitative variables were expressed as frequencies and percentages, and quantitative variables as means with standard deviations or medians with interquartile ranges, as appropriate. Group comparisons used Chi-square or Fisher's exact tests for categorical variables and the Mann–Whitney U test for continuous variables. Statistical significance was set at p≤0.05.

Time-to-event analyses were conducted separately at FU1 and FU2, with follow-up time defined from each visit to the first occurrence of the outcome within the subsequent observation period. PIF was treated as a time-updated exposure, measured at each visit. Separate Cox models were fitted for FU1 and FU2, allowing participants to contribute to both analyses while avoiding within-subject correlation within a single model.

Multivariable Cox proportional hazards models were used to evaluate time to first exacerbation and hospitalisation, including age, sex, smoking status, post-bronchodilator FEV_1_ (% predicted), FVC, inspiratory capacity, inhaler type and COPD Assessment Test (CAT) score as covariates. Model assumptions were assessed and verified prior to interpretation.

To assess whether the association between PIF and clinical outcomes differed by inhaler device type, interaction terms between PIF status and device class were additionally included in regression models for each outcome at FU1. Logistic regression was used for these analyses, as they were conducted as exploratory cross-sectional assessments at a single time-point.

## Results

A total of 415 participants were enrolled in the study, with 384 (92.5%) completing FU1 and 377 (90.8%) completing FU2, indicating high retention and minimal loss to follow-up. The mean age of the cohort was approximately 70 years.

### Suboptimal PIF and inhaler use

As defined in the Methods section, suboptimal PIF has different clinical implications depending on device type. For DPIs (R1–R5), it reflects insufficient inspiratory flow (“too slow”), whereas for pMDIs/SMIs (R0), it is excessive inspiratory flow (“too fast”). Unless otherwise specified, results refer to typical PIF measured at the resistance of the prescribed inhaler.

At FU1, 245 participants (63.8% of the cohort) were using a DPI, 71 (18.5%) an SMI and 68 (17.7%) a pMDI, as shown in table 1.

Among DPIs (R1–R5, n=245), 67% of participants (n=165) consistently achieved optimal PIF (can and will do), 22% (n=54) were capable but did not regularly do so (can, but won't do) and 11% (n=26) were unable to achieve the required PIF (cannot do) (figure 1). Variation in performance was also observed by resistance level at FU1 (figure 2).

**FIGURE 1:**
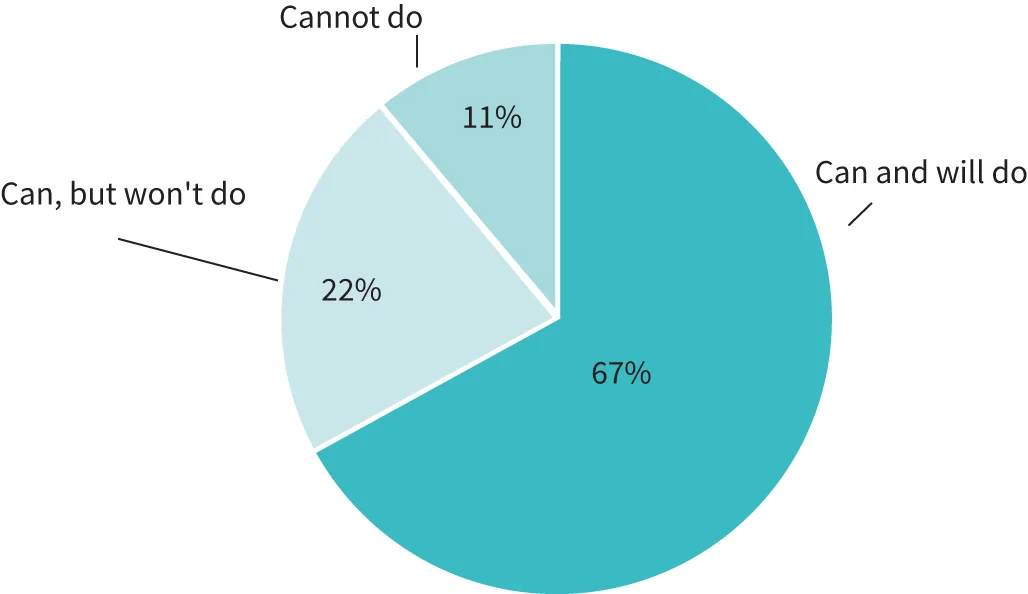
Proportion of participants using dry powder inhalers at follow-up (n=245), categorised into “can and will do”, “can, but won't do” and “cannot do” based on typical *versus* maximal peak inspiratory flow measurements.

**FIGURE 2:**
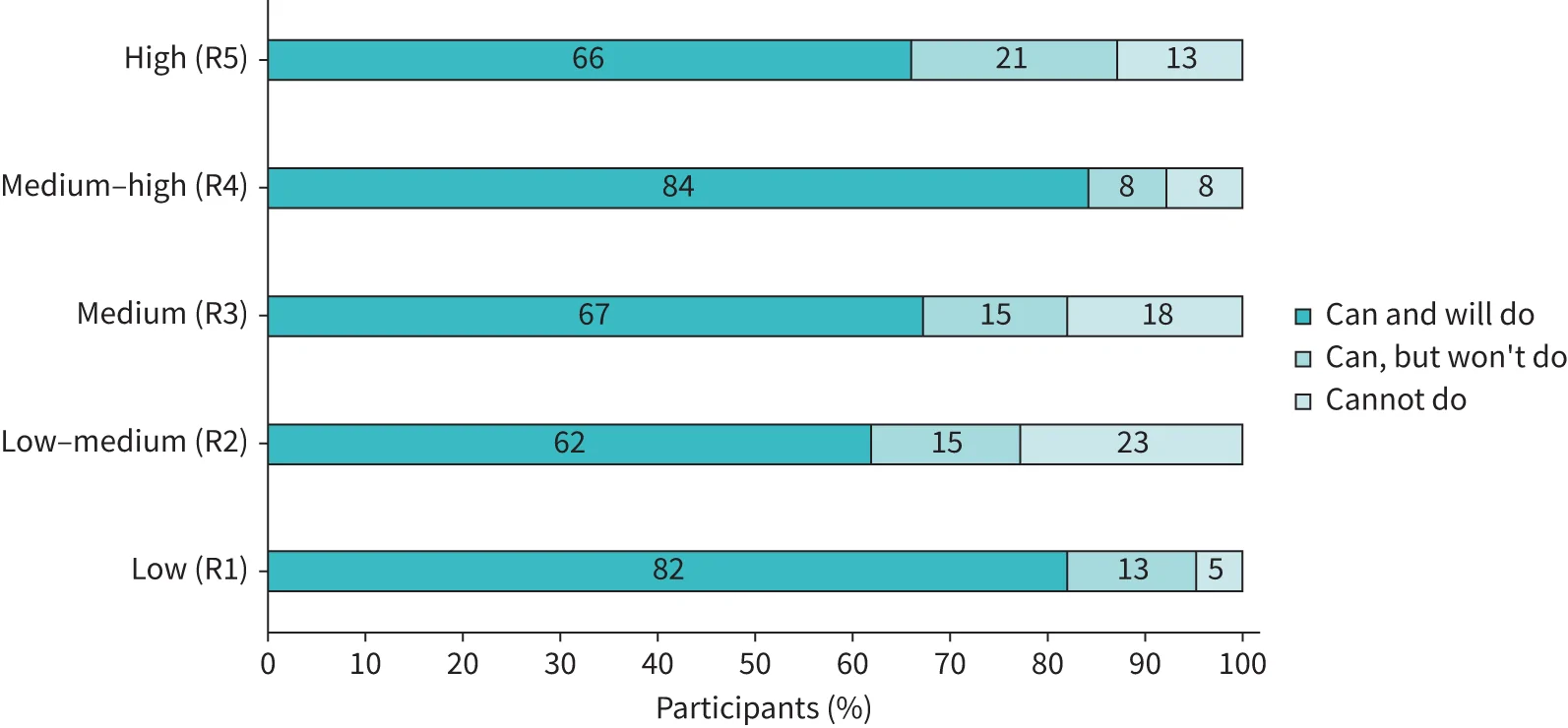
Distribution of inhalation performance categories across dry powder inhaler resistance levels (R1–R5) at follow-up 1 (n=245).

The distribution of optimal and suboptimal PIF varied by inhaler resistance, reflecting device type. At FU1, suboptimal PIF was most common with low-resistance inhalers (R0, 45.1%), where it reflected excessive inspiratory flow (>60 L·min^−1^, inappropriate for pMDI/SMI use). Suboptimal PIF was also observed with medium-resistance DPIs (R2, 30.1%) and was least common in high-resistance DPIs (R5, 3.5%). This pattern remained consistent at FU2 (table 2).

Across the full cohort (n=384), 113 participants (29.4%) had suboptimal PIF at FU1, based on typical inspiratory flow falling below or above the device-specific threshold for their prescribed inhaler's resistance (*i.e.* DPIs: R1–R5; pMDIs/SMIs: R0). By FU2 (n=377), 108 participants (28.6%) exhibited suboptimal PIF based on their typical measurements. Among those with suboptimal PIF, the proportion of female participants increased from 25.7% (n=29) at FU1 to 33.3% (n=36) at FU2 (p<0.001) and this group also consistently included a higher proportion of current smokers, with 24.7% (n=28) at FU1 and 25% (n=27) at FU2 (p<0.001).

### Suboptimal PIF and lung function

At FU1, participants with suboptimal PIF values (too low for DPIs or too fast for pMDIs/SMIs) showed lower mean pre-bronchodilator FEV_1_ (30.5±6.7% pred *versus* 38.6±7.7% pred, p=0.05) and actual measured FVC (1.55±1.20 L *versus* 2.00±1.70 L, p=0.05) compared to those with optimal PIF.

Post-bronchodilator differences were also observed, with the suboptimal PIF group showing reduced FEV_1_ (32.6±5.1% pred *versus* 40.6±7.5% pred, p=0.06), actual measured FVC (1.63±1.37 L *versus* 2.11±1.72 L, p=0.04) and inspiratory capacity, recorded separately during spirometry (0.88±0.90 L *versus* 1.00±0.70 L, p=0.04).

By FU2, the suboptimal PIF group continued to exhibit lower pre-bronchodilator FEV_1_ (33.7±9.1% pred *versus* 45.7±9.2% pred, p=0.01), post-bronchodilator FEV_1_ (36.5±9.4% pred *versus* 46.8±8.1% pred, p=0.04) and inspiratory capacity (0.76±0.90 L *versus* 1.07±0.80 L, p=0.02).

### Suboptimal PIF and disease exacerbations

Exacerbation rates differed according to typical PIF status (as defined above) at both FU1 and FU2. Moderate exacerbations, defined as those requiring antibiotics or oral steroids, showed no significant difference between participants with optimal and suboptimal PIF at FU1 (p=0.32). However, by FU2, moderate exacerbations were significantly more common in the suboptimal PIF group (p=0.03). Severe exacerbations, defined as those requiring emergency room visits or hospitalisation for more than 24 h, were also more frequent in the suboptimal PIF group by FU2 (p=0.01) (table 3).

**TABLE 3:** Comparison of demographics and clinical characteristics between participants with optimal and suboptimal peak inspiratory flow (PIF) status at follow-up 1 (FU1) and follow-up 2 (FU2)

	FU1					FU2		
	Total number of participants N=384 (100%)	Optimal PIF n=271 (70.5%)	Suboptimal PIF n=113 (29.4%)	p-value	Total number of participants N=377 (100%)	Optimal PIF n=269 (71.4%)	Suboptimal PIF n=108 (28.6%)	p-value
**Demographics**								
Age, mean±sd	70±7.6	70±8.9	69±11		70±8.7	71±9.5	70±8.5	
Sex				<0.001				<0.001
Female, n (%)	96 (25)	67 (24.6)	29 (25.6)		76 (20.2)	40 (14.8)	36 (33.3)	
Male, n (%)	288 (75)	205 (75.4)	83 (73.4)		301 (79.8)	229 (85.2)	72 (66.7)	
Smoking status				<0.001				<0.001
Current smoker, n (%)	96 (25)	68 (25.2)	28 (24.7)		107 (28.4)	82 (30.5)	25 (23)	
Former smoker, n (%)	288 (75)	202 (74.8)	86 (76.1)		270 (71.6)	187 (69.5)	83 (76)	
**Clinic characteristics**								
Pulmonary function tests								
Pre-bronchodilator values								
FVC (L), mean±sd	1.87±1.3	2.00±1.7	1.55±1.2	0.05	1.96±1.2	2.36±1.6	1.75±1.2	0.04
FEV_1_ (L), mean±sd	1.005±0.8	1.07±0.7	0.86±0.7	0.18	1.008±0.7	1.31±0.8	0.93±0.7	0.06
FEV_1_ (% pred), mean±sd	36.03±6.5	38.55±7.7	30.53±6.7	0.05	36.89±8.6	45.66±9.2	33.65±9.1	0.01
FEV_1_/FVC (%), mean±sd	31.92± 8.6	34.20±7.8	27.04±9.3	0.14	32.36±7.4	40.04±7.1	29.53±8.1	0.05
Post-bronchodilator values								
FVC (L), mean±sd	1.97±1.6	2.11±1.72	1.63±1.4	0.04	2.02±1.7	2.38±1.7	1.83±1.7	0.09
FEV_1_ (L), mean±sd	1.06±0.8	1.23±0.9	0.93±0.8	0.08	1.10±0.8	1.32±0.9	0.99±0.9	0.09
FEV_1_ (% pred), mean±sd	37.84±9.9	40.57±7.5	32.55±5.1	0.06	40.5±6.9	46.82±8.1	36.45±9.4	0.04
FEV_1_/FVC (%), mean±sd	32.76±8.3	35.27±7.5	27.28±9.1	0.07	35.7±7.6	39.87±7.7	31.88±8.6	0.06
Inspiratory capacity (L), mean±sd	0.90±0.8	1.00±0.7	0.88±0.9	0.04	0.81±0.9	1.07±0.8	0.76±0.9	0.02
Symptoms (CAT score), mean±sd	11.1±8.7	12.21±8.1	10.2±8.8	0.15	10.1±8.5	11.9±7.7	9.59±8.6	0.06
Exacerbations								
Moderate (acute courses of antibiotics or oral steroids for COPD)								
0, n (%)	303 (78.9)	216 (79.7)	87 (77.8)	0.32	265 (70.3)	192 (71.4)	73 (67.6)	0.03
1, n (%)	40 (10.4)	28 (10.3)	12 (10.6)		67 (17.8)	49 (18.2)	18 (16.3)	
2, n (%)	25 (6.5)	15 (5.4)	10 (8.8)		31 (8.2)	17 (6.3)	14 (13)	
3+, n (%)	16 (4.2)	12 (4.3)	4 (3.5)		14 (3.7)	11 (4)	3 (3.2)	
Severe (emergency room attendance or hospitalisation for >24 h)								
0, n (%)	313 (81.5)	225 (83)	88 (77.9)	0.13	317 (84)	241 (89.5)	76 (70.6)	0.01
1, n (%)	48 (12.5)	32 (11.9)	16 (14.1)		36 (9.5)	15 (5.6)	21 (18.6)	
2, n (%)	15 (3.9)	9 (3.3)	6 (5.3)		17 (4.5)	8 (3)	9 (8.7)	
3+, n (%)	8 (2.1)	5 (2)	3 (2.6)		7 (1.8)	5 (1.9)	2 (2.1)	

Values are presented as mean± sd or number (percentage), as appropriate. p-values were calculated using Chi-square tests (or Fisher's exact test when expected counts were <5) for categorical variables and a t-test or Mann–Whitney U test for continuous variables. Percentages in the total column are based on the overall cohort; percentages for optimal and suboptimal PIF groups are calculated within each group (column-wise). p-values represent comparisons between participants with optimal and suboptimal PIF within each follow-up visit (FU1 and FU2). FEV_1_: forced expiratory volume in 1 s; FVC: forced vital capacity.

In regression analyses conducted at FU1 and FU2 using typical PIF values, suboptimal PIF was consistently associated with an increased risk of exacerbations after adjustment for demographic factors, lung function characteristics and inhaler type (figure 3a, b).

**FIGURE 3:**
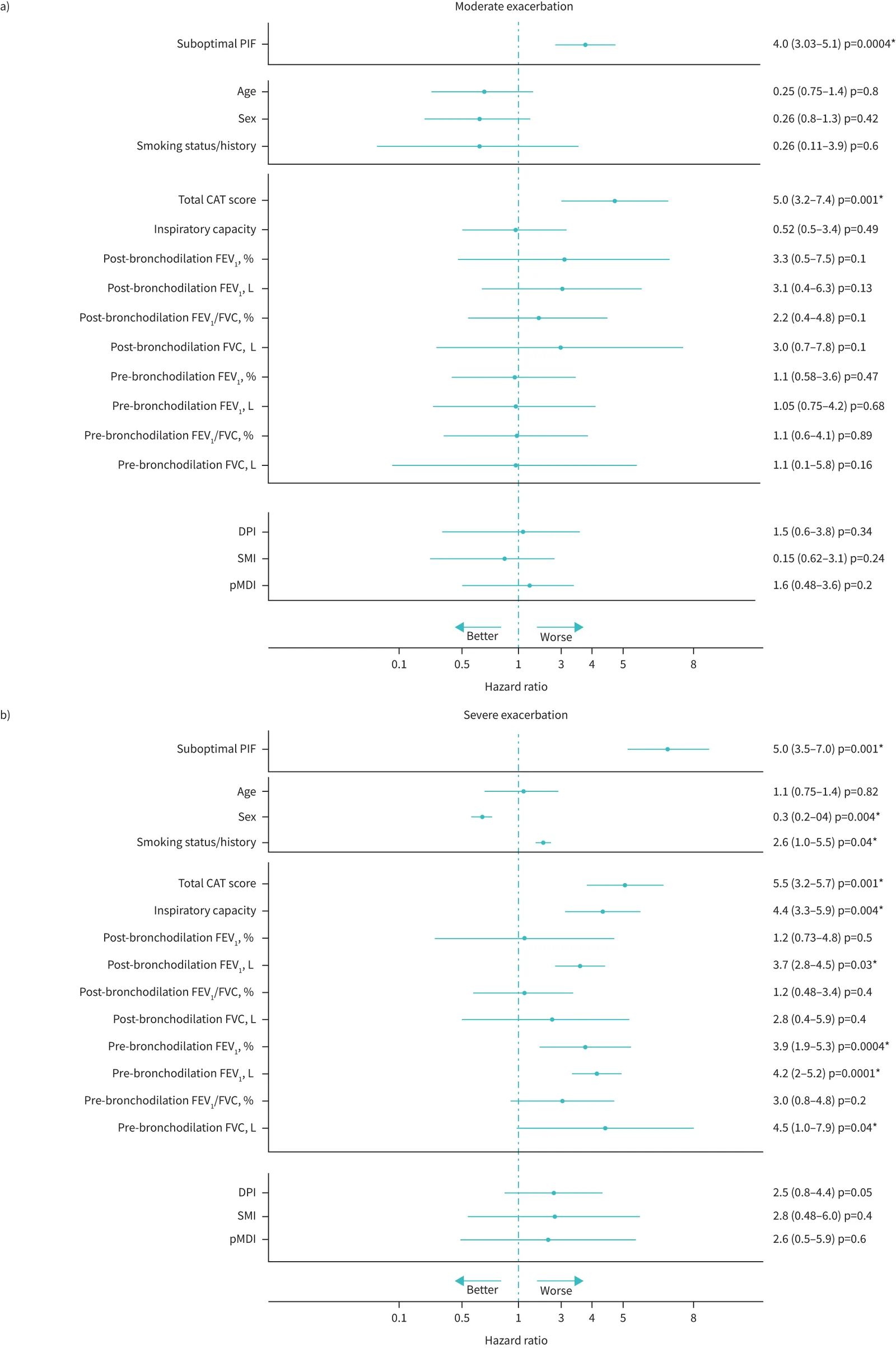
Hazard ratios (HRs) for a) moderate and b) severe exacerbations at follow-up 1 (FU1) and 2 (FU2) in participants with optimal and suboptimal maximal peak inspiratory flow (PIF). Points represent HRs and horizontal lines indicate 95% confidence intervals from multivariable Cox proportional hazards models. The dashed vertical line represents HR=1. Statistical significance was defined as p≤0.05 (*). Models were fitted separately at FU1 and FU2, with follow-up time defined from each visit. Suboptimal PIF was defined as a typical inspiratory flow below the optimal threshold for dry powder inhalers (DPIs) or above the optimal threshold for pressurised metered-dose inhalers (pMDIs)/soft mist inhalers (SMIs). Low- and high-resistance PIF measurements were not collected at FU2; therefore, only typical PIF was used to ensure consistency across follow-up visits. Forced expiratory volume in 1 s (FEV_1_) values are expressed as % predicted. FVC: forced vital capacity.

For moderate exacerbations, participants with suboptimal PIF had a significantly higher risk (hazard ratio (HR) 4, 95% CI 3.03–5.1; p=0.0004). Higher CAT scores were also associated with increased risk (HR 5.0, 95% CI 3.2–7.4; p=0.001). Demographic factors such as age, sex and smoking status were not significant predictors (figure 3a).

For severe exacerbations, suboptimal PIF remained a predictor (HR 5.0, 95% CI 3.5–7.0; p=0.001). Higher CAT scores were again associated with increased risk (HR 5.5, 95% CI 3.2–5.7; p=0.001).

Several lung function parameters were significantly associated with exacerbations, including reduced inspiratory capacity (HR 4.4, 95% CI 3.3–5.9; p=0.004), lower post-bronchodilator FEV_1_ (L) (HR 3.7, 95% CI 2.8–4.5; p=0.03) and lower pre-bronchodilator FEV_1_ (both % predicted and L) (HR 3.9, 95% CI 1.9–5.3; p=0.0004 and HR 4.2, 95% CI 2.0–5.2; p=0.0001, respectively).

Smoking status (HR 2.6, 95% CI 1.0–5.5; p=0.04) and sex (HR 0.3, 95% CI 0.2–0.4; p=0.004) were also significantly associated with severe exacerbations, while age was not.

Inhaler type showed some variation in estimates, with DPI users demonstrating a borderline increase in risk (p=0.05), whereas SMI and pMDI were not associated (figure 3b).

To explore whether the association between PIF and clinical outcomes differed by inhaler device type, cross-sectional interaction analyses were performed at FU1 using logistic regression models. Across moderate exacerbations, severe exacerbations and hospitalisations, suboptimal PIF showed a consistent trend towards increased risk. No significant interaction between PIF status and device class was observed (all p>0.5) and effect estimates for suboptimal PIF were directionally consistent across device types (table S1 and figure S1).

### Suboptimal PIF and hospitalisation

Hospitalisation risk was evaluated in relation to typical PIF status, with regression analyses conducted separately at FU1 and FU2.

At FU1, there was no significant difference in hospitalisation risk between participants with optimal and suboptimal PIF (HR 1.23, 95% CI 0.7–1.5) and no clear associations were observed for demographic or lung function variables. However, by FU2, increased hospitalisation rates among participants with suboptimal PIF were observed (HR 1.5, 95% CI 1.1–1.9; p=0.05). Key lung function variables, including post-bronchodilator FEV_1_ (% predicted) and inspiratory capacity, also showed some associations with hospitalisation (figure 4).

**FIGURE 4:**
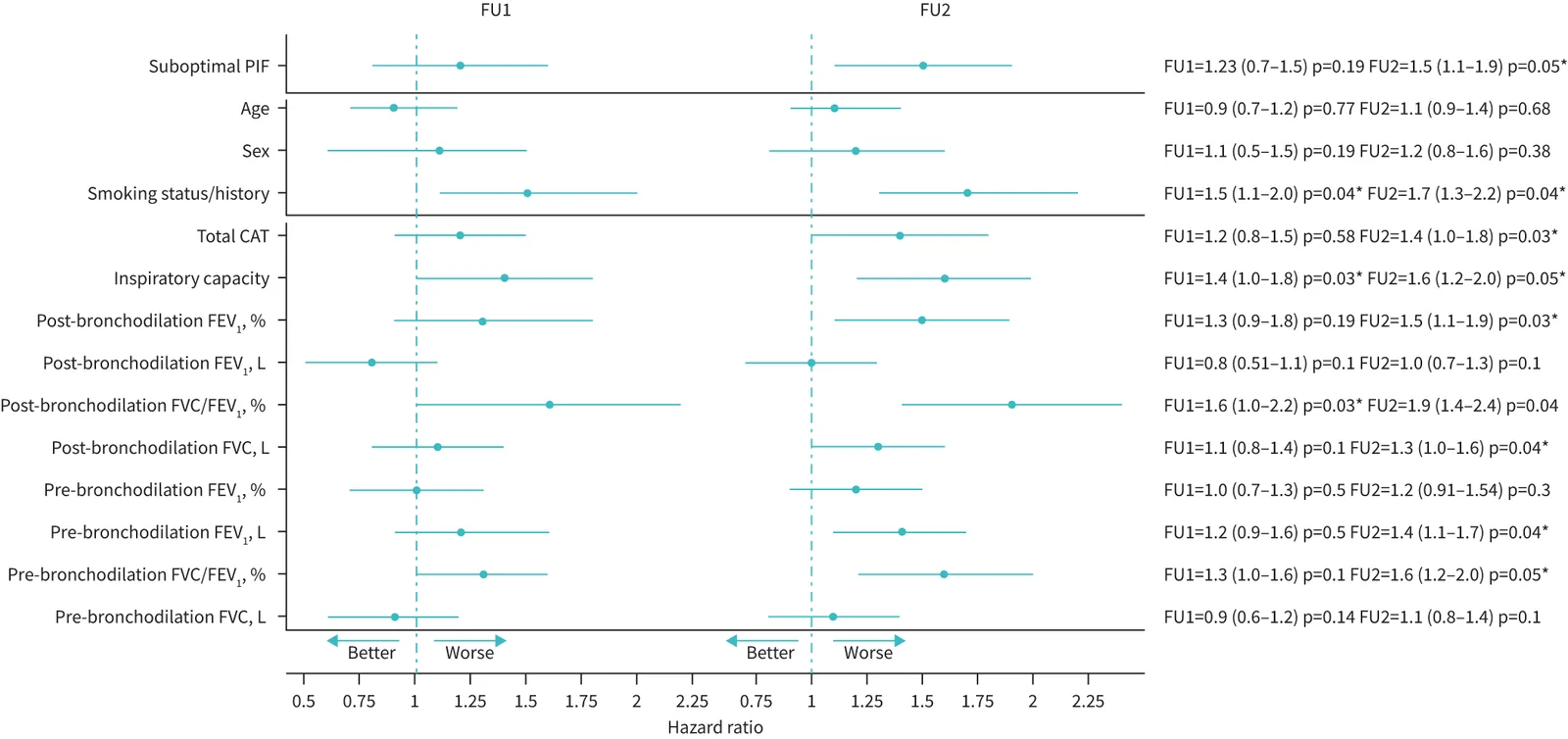
Hospitalisation risk at follow-up 1 (FU1) and 2 (FU2) in participants with optimal and suboptimal peak inspiratory flow (PIF). Points represent hazard ratios (HRs) and horizontal lines indicate 95% confidence intervals. The dashed vertical line represents HR=1. Statistical significance was defined as p≤0.05 (*). Models were fitted separately at FU1 and FU2, with follow-up time defined from each visit. Suboptimal PIF was defined as a typical inspiratory flow below the optimal threshold for dry powder inhalers or above the optimal threshold for pressurised metered-dose inhalers/soft mist inhalers. Low- and high-resistance PIF measurements were not collected at FU2; therefore, only typical PIF was used to ensure consistency across follow-up visits. Forced expiratory volume in 1 s (FEV_1_) values are expressed as % predicted. CAT: COPD Assessment Test; FVC: forced vital capacity.

### Suboptimal PIF and disease burden

CAT scores were slightly lower in the suboptimal typical PIF group at both follow-ups (FU1: 10.2±8.8 *versus* 12.2±8.1, p=0.15; FU2: 9.6±8.6 *versus* 12.0±7.7, p=0.06), although these differences were not statistically significant (table 3).

Participants with suboptimal typical PIF were more likely to require additional therapies, indicating greater treatment use. By FU2, this group demonstrated higher use of short-acting beta-agonists (SABA) (20%, n=7/35) and oral corticosteroids (8%, n=3/38). Triple therapy (inhaled corticosteroid/long-acting beta-agonist/long-acting muscarinic antagonist) was also more prevalent among those with suboptimal typical PIF, with 28% (n=10/35) at FU1 and 23.6% (n=9/38) at FU2 (table 4).

**TABLE 4:** Comparison of medication use between participants with optimal and suboptimal peak inspiratory flow (PIF) status at follow-up 1 (FU1) and follow-up 2 (FU2)

	FU1					FU2		
	Total number of participants N=126 (100%)	Optimal PIF n=91 (72.2%)	Suboptimal PIF n=35 (27.8%)	p-value	Total No of Participants N=91 (100%)	Optimal PIF n=54 (59.3%)	Suboptimal PIF n=37 (40.7%)	p-value
**Medications, n (%)**								
SABA	23 (18)	20 (22)	3 (8.6)	0.004	29 (32)	19 (31.5)	10 (26.3)	0.002
SAMA	7 (6)	6 (6.6)	1 (3)		9 (10)	5 (9)	4 (11)	
LABA	7 (6)	6 (6.6)	1 (3)		3 (3)	2 (4)	1 (3)	
LAMA	15 (12)	14 (15.4)	1 (3)		1 (1)	1 (2)	0 (0)	
ICS	18 (14)	16 (17.6)	2 (5.7)		5 (5)	1 (2)	4 (10.5)	
ICS/LAMA	8 (9)	7 (7.7)	1 (3)		6 (7)	3 (6)	3 (8)	
LABA/LAMA	7 (5.6)	3 (3.3)	4 (11.4)		2 (2)	1 (2)	1 (3)	
ICS/LABA/LAMA	14 (11)	6 (6.6)	8 (23)	0.04	11 (12)	2 (4)	9 (23.7)	0.05
OCS	1 (1)	0 (0)	1 (3)		5 (5)	2 (4)	3 (8)	0.02
Theophylline	7 (5)	5 (5.5)	2 (6)		1 (1)	1 (2)	0 (0)	
Macrolides	3 (3)	2 (2.2)	1 (3)		2 (2)	1 (2)	1 (3)	
Antibiotics	3 (2.4)	2 (2.2)	1 (3)		2 (2)	1 (2)	1 (3)	
Mucolytics	8 (6.3)	1 (1.1)	7 (20)		6 (7)	3 (6)	3 (8)	
**Other treatments, n (%)**								
LTOT	3 (3)	2 (2.2)	1 (3)		2 (2)	1 (2)	1 (3)	
NIV	2 (2)	1 (1.1)	1 (3)		1 (1)	1 (2)	0 (0)	

Values are expressed as absolute (relative) frequencies. p-values (≤0.05) were calculated using Chi-square tests (or Fisher's exact test when expected frequencies were <5) for categorical variables and Mann–Whitney U or t-tests for continuous variables. p-values represent comparisons between optimal and suboptimal PIF groups within each follow-up visit. Medication data were available for a subset of participants at each follow-up visit. ICS: inhaled corticosteroids; LABA: long-acting beta-agonist; LAMA: long-acting muscarinic antagonist; LTOT: long-term oxygen therapy; NIV: noninvasive ventilation; OCS: oral corticosteroid; SABA: short-acting beta agonist; SAMA: short-acting muscarinic antagonist.

## Discussion

In this analysis, suboptimal PIF was considered as a unified exposure to reflect real-world inhaler use across device types. However, insufficient inspiratory flow in DPIs and excessive flow in pMDIs/SMIs represent distinct physiological and behavioural phenomena, and this approach may mask device-specific effects.

2This longitudinal analysis extends our previously published baseline findings [15], showing that suboptimal PIF is prevalent and associated with markers of disease severity and increased exacerbation risk in COPD. By incorporating follow-up data at 3 and 6 months, we demonstrate that suboptimal PIF persists over time and is independently associated with exacerbation risk after adjustment for traditional lung function measures.

Although multivariable adjustment was performed, suboptimal PIF may partly reflect underlying disease severity, impaired inspiratory capacity or other unmeasured factors such as inhaler technique, adherence or frailty. It should therefore be interpreted as a marker of increased risk rather than a direct causal driver of clinical outcomes. Nevertheless, as a marker of patient–device mismatch, suboptimal PIF may still have important clinical implications for inhaler selection and optimisation of treatment delivery.

Suboptimal PIF has different implications depending on inhaler type. For DPIs it reflects insufficient inspiratory effort (“too slow”), whereas for pMDIs and SMIs it indicates excessive inspiratory flow (“too fast”). At both follow-ups, around 30% of participants had suboptimal PIF at their prescribed device resistance, reinforcing that this is a stable and clinically meaningful marker. Importantly, women and current smokers were overrepresented in this group, suggesting patient characteristics that may warrant closer clinical attention.

These findings align with prior research suggesting that women and smokers may have respiratory profiles or physical characteristics linked to inspiratory capacity [19]. Clinically, these demographics, particularly sex and smoking status, should be considered when assessing PIF and selecting appropriate inhalers [20, 21]. In our study, participants with suboptimal PIF demonstrated lower lung function across follow-up visits. However, CAT scores did not differ significantly between groups, despite being numerically lower in the suboptimal PIF group [22, 23].

Consistent with these observations, regression analyses confirmed that suboptimal PIF was independently associated with both moderate and severe exacerbations. The magnitude of the observed HRs should be interpreted with caution. These estimates may partly reflect residual confounding and the strong association between suboptimal PIF and markers of disease severity, which are themselves closely linked to exacerbation risk.

Participants with suboptimal PIF had a fourfold higher risk of moderate exacerbations and an approximately fivefold higher likelihood of severe exacerbations. These associations persisted after adjusting for demographic factors, lung function and inhaler type, underscoring the independent clinical association of impaired PIF.

Importantly, while symptom burden (*e.g.* CAT score) is a recognised predictor of exacerbation risk, PIF provides complementary information by reflecting the patient's ability to effectively use their inhaler and achieve adequate drug delivery. In this context, suboptimal PIF may represent a modifiable, treatment-related factor with direct implications for inhaler selection and technique optimisation. This supports the potential clinical value of incorporating PIF assessment into routine care to identify patients at risk of suboptimal drug delivery and to guide personalised inhaler selection and targeted interventions.

In addition, interaction analyses did not identify evidence of a differential effect by device class, suggesting that the association between suboptimal PIF and clinical outcomes may be broadly similar across inhaler types despite differing underlying physiological mechanisms.

Suboptimal PIF may reflect not only device resistance but also improper inhalation technique and patient-related limitations [24]. Participants with suboptimal PIF were more likely to experience exacerbations [25]. These findings suggest that integrating inhaler technique training with PIF evaluation may help reduce exacerbation risk and improve disease control [26, 27]. This is relevant for those classified as “can, but won't do”, who may benefit from targeted education and technique correction. Identifying patients with suboptimal PIF can therefore support optimised therapy and improved clinical outcomes [28, 29].

Hospitalisation risk did not differ by PIF status at FU1, but was higher in participants with suboptimal PIF at FU2, suggesting that the association between impaired PIF and severe outcomes may become more apparent over time. Lung function parameters, including FEV_1_ (% predicted), FVC and inspiratory capacity, were also associated with hospitalisation at FU2. These findings reinforce the importance of longitudinal PIF monitoring in COPD management [30–33] and raise the question of whether suboptimal performance reflects physiological limitation or inhalation technique.

At FU1, 67% of participants achieved optimal PIF (“can and will do”), while 22% were capable but did not consistently do so (“can, but won't do”) and 11% were unable to generate sufficient PIF (“cannot do”), resulting in 33% with suboptimal performance. This proportion is consistent with findings from the PIFotal study, conducted in primary care, where 29% of participants failed to generate an optimal PIF during typical inhalation [17]. Importantly, PIFotal further distinguished between technique-related limitations (“can, but won't do”) and physiological limitations (“cannot do”), a framework that helps contextualise our results and suggests that many COPD patients may benefit from targeted inhalation training or reconsideration of device type.

Adherence and inhaler technique were not formally assessed at follow-up visits in our study, which represents an important limitation, as inhaler technique and adherence are likely to be strongly associated with both PIF performance and clinical outcomes, and may partially explain the observed associations. In particular, the “can, but won't do” subgroup may reflect suboptimal inhalation technique rather than physiological limitation. As such, suboptimal PIF likely captures a combination of physiological and behavioural factors, and its association with clinical outcomes should be interpreted in this context. The increased use of SABA and triple therapy among participants with suboptimal PIF may indicate a greater symptom burden or suboptimal medication delivery [34, 35], a pattern also noted in the PIFotal study [17].

This study has limitations. Although multivariable adjustment was performed, residual confounding cannot be excluded. Low-and high-resistance PIF measurements were not repeated at FU2, limiting interpretation. Medication adherence was not assessed, which may have influenced both PIF performance and clinical outcomes. Exacerbations were based on patient-reported data, which may introduce recall bias.

### Conclusions

Suboptimal PIF was associated with poorer lung function, increased exacerbations and hospitalisations, and greater treatment burden over time. These findings confirm that the clinical association of impaired PIF persists beyond a single time-point and support the value of incorporating PIF assessment into routine COPD care to guide inhaler selection and optimise patient outcomes.

## Supplementary material

**Please note:** supplementary material is not edited by the Editorial Office, and is uploaded as it has been supplied by the author. Supplementary material 00343-2026.SUPPLEMENT

## References

[1] Raherison C, Girodet PO. Epidemiology of COPD. Eur Respir Rev 2009; 18: 213–221. doi:10.1183/09059180.00003609 20956146

[2] Negewo NA, Gibson PG, McDonald VM. COPD and its comorbidities: impact, measurement and mechanisms. Respirology 2015; 20: 1160–1171. doi:10.1111/resp.12642 26374280

[3] Uwagboe I, Adcock IM, Lo Bello F, et al. New drugs under development for COPD. Minerva Med 2022; 113: 471–496. doi:10.23736/S0026-4806.22.08024-7 35142480

[4] Leving MT, Kocks J, Bosnic-Anticevich S, et al. Relationship between peak inspiratory flow and patient and disease characteristics in individuals with COPD–a systematic scoping review. Biomedicines 2022; 10: 458. doi:10.3390/biomedicines10020458 PMC8962311 35203667

[5] Mahler DA, Watz H, Emerson-Stadler R, et al. Clinical implications of peak inspiratory flow in COPD: *post hoc* analyses of the TRONARTO study. Int J Chron Obstruct Pulmon Dis 2023; 18: 1729–1740. doi:10.2147/COPD.S404243 PMC10432827 37599896

[6] Ohar JA, Ferguson GT, Mahler DA, et al. Measuring peak inspiratory flow in patients with chronic obstructive pulmonary disease. Int J Chron Obstruct Pulmon Dis 2022; 17: 79–92. doi:10.2147/COPD.S319511 PMC8747625 35023914

[7] Anderson M, Collison K, Drummond MB, et al. Peak inspiratory flow rate in COPD: an analysis of clinical trial and real-world data. Int J Chron Obstruct Pulmon Dis 2021; 16: 933–943. doi:10.2147/COPD.S291554 PMC8055277 33883890

[8] Leving MT, van Boven JFM, Bosnic-Anticevich SZ, et al. Suboptimal peak inspiratory flow and critical inhalation errors are associated with higher COPD-related healthcare costs. Int J Chron Obstruct Pulmon Dis 2022; 17: 2401–2415. doi:10.2147/COPD.S380736 PMC9521797 36185173

[9] Terzano C, Oriolo F. Lung characteristics in elderly males and females patients with COPD: differences and optimal use of dry powder inhalers (DPIs). Eur Rev Med Pharmacol Sci 2017; 21: 2708–2716. 28678314

[10] Broeders ME, Molema J, Hop WC, et al. Inhalation profiles in asthmatics and COPD patients: reproducibility and effect of instruction. J Aerosol Med 2003; 16: 131–141. doi:10.1089/089426803321919898 12823907

[11] Jõgi R, Mattila L, Vahteristo M, et al. Inspiratory flow parameters through dry powder inhalers in healthy volunteers and patients with chronic obstructive pulmonary disease (COPD): device resistance does not limit use in COPD. Int J Chron Obstruct Pulmon Dis 2021; 16: 1193–1201. doi:10.2147/COPD.S298514 PMC8096421 33958863

[12] Sanchez-Guerrero J, Guerlain J, Samaha S, et al. Upper airway obstruction assessment: peak inspiratory flow and clinical COPD questionnaire. Clin Otolaryngol 2018; 43: 1303–1311. doi:10.1111/coa.13149 29797692

[13] Mollaoglu M, Fertelli TK, Tuncay FÖ. Fatigue and disability in elderly patients with chronic obstructive pulmonary disease (COPD). Arch Gerontol Geriatr 2011; 53: e93–e98. doi:10.1016/j.archger.2010.07.001 20705348

[14] Chetta A, Yorgancioglu A, Scuri M, et al. Inspiratory flow profile and usability of the NEXThaler, a multidose dry powder inhaler, in asthma and COPD. BMC Pulm Med 2021; 21: 65. doi:10.1186/s12890-021-01430-9 PMC7905605 33632183

[15] Perugini V, Rhee CK, Moon J-Y, et al. Assessment of peak inspiratory flow in patients with chronic obstructive pulmonary disease: a multicentre, observational, prospective, real-life study. BMJ Open Respir Res 2025; 12: e002408. doi:10.1136/bmjresp-2024-002408 PMC12481310 40998461

[16] Agusti A, Vogelmeier CF. GOLD 2024: a brief overview of key changes. J Bras Pneumol 2023; 49: e20230369. doi:10.36416/1806-3756/e20230369 PMC10760434 38126685

[17] Kocks J, Wouters H, Bosnic-Anticevich S, et al. Factors associated with health status and exacerbations in COPD maintenance therapy with dry powder inhalers. NPJ Prim Care Respir Med 2022; 32: 18. doi:10.1038/s41533-022-00282-y PMC9135702 35618739

[18] Vandenbroucke JP, von Elm E, Altman DG, et al. Strengthening the Reporting of Observational Studies in Epidemiology (STROBE): explanation and elaboration. Int J Surg 2014; 12: 1500–1524. doi:10.1016/j.ijsu.2014.07.014 25046751

[19] Roy A, Pleasants RA, Henderson AG, et al. Effect of physical position on peak inspiratory flow in stable COPD: an observational study. Chronic Obstr Pulm Dis 2024; 11: 174–186. doi:10.15326/jcopdf.2023.0460 PMC11075346 38236166

[20] Self TH, Cross LB, Nolan SE, et al. Gender differences in the use of peak flow meters and their effect on peak expiratory flow. Pharmacotherapy 2005; 25: 526–530. doi:10.1592/phco.25.4.526.61026 15977914

[21] Finch CK, Tolley E, James A, et al. Gender differences in peak flow meter use. Nurse Pract 2007; 32: 46–48. doi:10.1097/01.NPR.0000269474.11424.80 17476168

[22] Self TH, George CM, Wallace JL, et al. Incorrect use of peak flow meters: are you observing your patients? J Asthma 2014; 51: 566–572. doi:10.3109/02770903.2014.914218 24720711

[23] Moon JY, Kim SH, Kim Y, et al. Suboptimal peak inspiratory flow rate in dry-powder inhaler users for chronic obstructive pulmonary disease in Korea. Pulm Pharmacol Ther 2024; 85: 102298. doi:10.1016/j.pupt.2024.102298 38604404

[24] Duarte AG, Tung L, Zhang W, et al. Spirometry measurement of peak inspiratory flow identifies suboptimal use of dry powder inhalers in ambulatory patients with COPD. Chronic Obstr Pulm Dis 2019; 6: 246–255. doi:10.15326/jcopdf.6.3.2018.0163 PMC6872217 31189057

[25] Loh CH, Peters SP, Lovings TM, et al. Suboptimal inspiratory flow rates are associated with chronic obstructive pulmonary disease and all-cause readmissions. Ann Am Thorac Soc 2017; 14: 1305–1311. doi:10.1513/AnnalsATS.201611-903OC 28406710

[26] Ding N, Zhang W, Wang Z, et al. Prevalence and associated factors of suboptimal daily peak inspiratory flow and technique misuse of dry powder inhalers in outpatients with stable chronic airway diseases. Int J Chron Obstruct Pulmon Dis 2021; 16: 1913–1924. doi:10.2147/COPD.S311178 PMC8236256 34188467

[27] González-Montaos A, Pazos-Area L, Represas-Represas C, et al. Suboptimal peak inspiratory flow in patients hospitalized for COPD exacerbation: prevalence and predictive factors. J Aerosol Med Pulm Drug Deliv 2024; 37: 225–231. doi:10.1089/jamp.2024.0002 PMC11502629 39388689

[28] Sanders MJ. Guiding inspiratory flow: development of the in-check DIAL G16, a tool for improving inhaler technique. Pulm Med 2017; 2017: 1495867. doi:10.1155/2017/1495867 PMC5733915 29348936

[29] Mahler DA. The role of inspiratory flow in selection and use of inhaled therapy for patients with chronic obstructive pulmonary disease. Respir Med 2020; 161: 105857. doi:10.1016/j.rmed.2019.105857 32056720

[30] Hira D, Okuda T, Ichihashi M, et al. Influence of peak inspiratory flow rates and pressure drops on inhalation performance of dry powder inhalers. Chem Pharm Bull (Tokyo) 2012; 60: 341–347. doi:10.1248/cpb.60.341 22382414

[31] Weiner P, Weiner M. Inspiratory muscle training may increase peak inspiratory flow in chronic obstructive pulmonary disease. Respiration 2006; 73: 151–156. doi:10.1159/000088095 16155355

[32] Grandmaison G, Grobéty T, Vaucher J, et al. Prevalence of critical errors and insufficient peak inspiratory flow in patients hospitalized with COPD in a department of general internal medicine: a cross-sectional study. Chronic Obstr Pulm Dis 2024; 11: 406–415. doi:10.15326/jcopdf.2024.0505 PMC11363973 38901836

[33] Clark B, Wells BJ, Saha AK, et al. Low peak inspiratory flow rates are common among COPD inpatients and are associated with increased healthcare resource utilization: a retrospective cohort study. Int J Chron Obstruct Pulmon Dis 2022; 17: 1483–1494. doi:10.2147/COPD.S355772 PMC9250781 35791340

[34] Meng W, Xiong R, Zhao Z, et al. Suboptimal peak inspiratory flow rate: a noticeable risk factor for inhaler concordance in patients with chronic airway diseases. BMJ Open Respir Res 2024; 11: e001981. doi:10.1136/bmjresp-2023-001981 PMC11033634 38642917

[35] Pankovitch S, Frohlich M, AlOthman B, et al. Peak inspiratory flow and inhaler prescription strategies in a specialized COPD clinical program: a real-world observational study. Chest 2025; 167: 736–745. doi:10.1016/j.chest.2024.09.031 PMC11882739 39384100

