# Supplementary material for: Suboptimal peak inspiratory flow, COPD exacerbations, hospitalisations and disease burden: a longitudinal follow-up in a multicentre real-world study
Source: ERJ Open Res. 2026 Nov 9;12(6):00343-2026. doi: 10.1183/23120541.00343-2026 (PMC13750554; doi:10.1183/23120541.00343-2026)
Supplement: Supplementary file 1 [file 00343-2026.SUPPLEMENT.pdf]

Supplementary Table S1. Association between suboptimal peak inspiratory flow (PIF), inhaler device class, and clinical outcomes at follow-up 1 (FU1).

Moderate exacerbations (FU1)

	**OR (95% CI)**	***p*-value**
Suboptimal PIF	2.38 (0.85-6.69)	0.1
Device	0.76 (0.26-2.20)	0.61
Interaction	0.61 (0.10-3.68)	0.59

Severe exacerbations (FU1)

	**OR (95% CI)**	***p*-value**
Suboptimal PIF	2.47 (0.59-10.32)	0.21
Device	0.33 (0.04-2.76)	0.31
Interaction	1.98 (0.12-33.13)	0.64

Hospitalisation (FU1)

	**OR (95% CI)**	***p*-value**
Suboptimal PIF	1.99 (0.59-6.68)	0.27
Device	0.39 (0.08-1.81)	0.23
Interaction	1.88 (0.21-16.79)	0.57

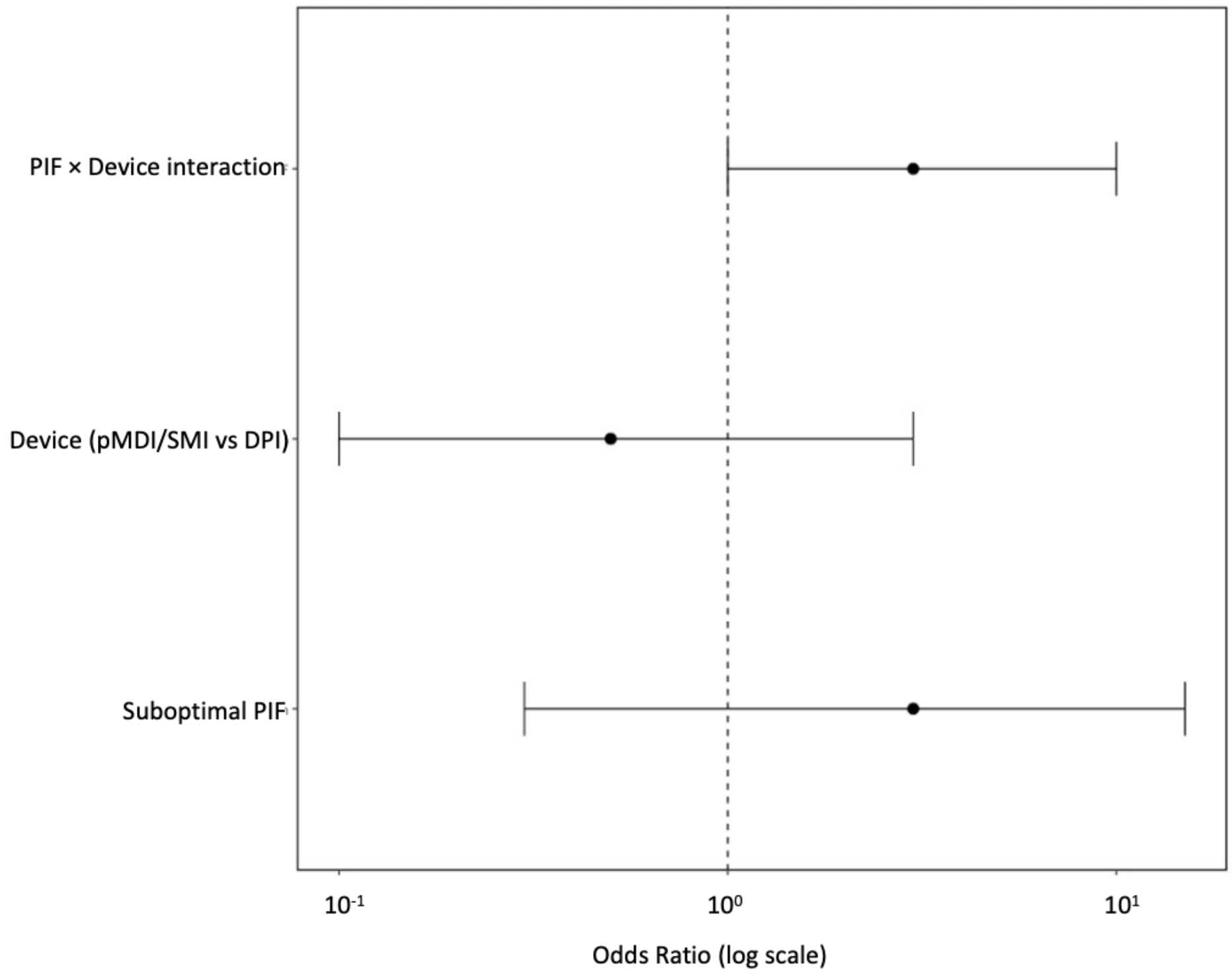

Definitions: Suboptimal PIF refers to PIF below the optimal threshold for DPIs and above the optimal threshold for pMDI/SMI devices. Device refers to inhaler class (pMDI/SMI vs DPI). Interaction represents the statistical interaction term between PIF status and device class.

Supplementary Figure S1. Association between suboptimal peak inspiratory flow (PIF) and severe exacerbations at follow-up 1 (FU1).

Forest plot showing odds ratios (ORs) and 95% confidence intervals (CIs) from logistic regression models including PIF status, inhaler device class (pMDI/SMI vs DPI), and their interaction term. Estimates are presented on a logarithmic scale.
